# Haptoglobin Genotype and Outcome after Subarachnoid Haemorrhage: New Insights from a Meta-Analysis

**DOI:** 10.1155/2017/6747940

**Published:** 2017-09-26

**Authors:** Ben Gaastra, James Glazier, Diederik Bulters, Ian Galea

**Affiliations:** ^1^Wessex Neurological Centre, University Hospital Southampton, Southampton SO21 2AS, UK; ^2^Clinical Neurosciences, Clinical and Experimental Sciences, Faculty of Medicine, University of Southampton, Southampton SO16 6YD, UK

## Abstract

Haptoglobin (Hp) is a plasma protein involved in clearing extracellular haemoglobin and regulating inflammation; it exists as two genetic variants (Hp1 and Hp2). In a meta-analysis of six published studies, we confirm that Hp genotype affects short-term outcome (cerebral vasospasm and/or delayed cerebral ischemia) after subarachnoid haemorrhage (SAH) but not long-term outcome (Glasgow Outcome Score and modified Rankin Scale between one and three months). A closer examination of the heterozygous group revealed that the short-term outcome of Hp2-1 individuals clustered with that of Hp1-1 and not Hp2-2, suggesting that the presence of one Hp1 allele was sufficient to confer protection. Since the presence of the Hp dimer is the only common feature between Hp1-1 and Hp2-1 individuals, the absence of this Hp moiety is most likely to underlie vasospasm in Hp2-2 individuals. These results have implications for prognosis after SAH and will inform further research into Hp-based mechanism of action and treatment.

## 1. Introduction

Haptoglobin (Hp) is an acute phase protein that binds to extracellular haemoglobin (Hb) with very high affinity. The resulting Hp-Hb complex is scavenged via CD163 expressed by cells of myeloid lineage [1]. There are two Hp alleles, Hp1 and Hp2. Hp2 is a longer protein which arose by an intragenic duplication event affecting exons 3 and 4 in the Hp1 gene. Recent data suggests that this happened at some point very early in human evolution, followed by recurring exonic deletions to reestablish modern Hp1 [2]. Individuals can express one of three Hp genotypes: Hp1-1, Hp2-1, and Hp2-2. The alpha chain of Hp has one cysteine residue in Hp1 but two cysteine residues in Hp2. These cysteine residues can form intermolecular disulphide bonds to give rise to Hp molecules of different sizes [3]. Hp1-1 homozygotes only form Hp dimers, Hp2-2 homozygotes only form higher-order Hp polymers, while Hp2-1 heterozygous individuals form both Hp dimer and higher-order Hp polymers. In Hp2-2 homozygotes, polymers are cyclic (i.e., Hp(*α*2*β*)_*n*_ where *n* = 3 and above). In Hp2-1 heterozygotes, linear polymers form since polymer growth is arrested by Hp1 at both ends (i.e., Hp(*α*1*β*)_2_(*α*2*β*)_*n*_ where *n* = 0 and above) [4].

Aneurysmal subarachnoid haemorrhage (aSAH) carries substantial morbidity and mortality. A common and serious complication of aSAH is that of cerebral vasospasm (CV). Prolonged or pronounced vasoconstriction of major cerebral blood vessels can lead to delayed cerebral ischaemia (DCI), which occurs in up to 30% of individuals who survive aSAH, manifesting as new focal neurological signs and/or deterioration in level of consciousness. Together, CV and DCI contribute to short-term outcome, by increasing short-term morbidity, hospital stay, and costs [5]. Longer-term outcome and functional status after aSAH are typically assessed using the modified Rankin Scale (mRS) or the Glasgow Outcome Scale (GOS).

Hp alleles profoundly affect outcome after intracranial haemorrhage, such as aSAH; Hp2 confers a poorer prognosis, with odds ratios of up to 4 being reported [6]. The underlying mechanism remains to be established. There are three potential biological mechanisms to explain this phenomenon: difference in Hp expression, Hp function, and Hp-Hb complex size. There is agreement that serum Hp expression is highest in Hp1-1 individuals, intermediate in Hp2-1, and lowest in Hp2-2 individuals (Table 1). With respect to functional aspects of Hp relevant to SAH, these include affinity of Hp binding to Hb, Hb binding capacity of Hp, protection from Hb's redox toxicity, affinity to CD163, CD163-mediated uptake, and effects on inflammation (Table 1). There is a lack of agreement as to whether Hp1 and Hp2 differ with respect to some of these aspects, and in which direction, as reviewed in Table 1. The third potential mechanism relates to the fact that the dimer produced by Hp1-1 and Hp2-1 individuals is smaller than other higher-order polymers produced by Hp2-2 individuals. This may be important since solute drainage from the brain along the glymphatic pathway has a size selectivity [7]. Drainage of Hp-Hb complexes from the brain to the circulation may be important since CD163 binding sites are reduced and saturated in the brain [8, 9].

The outcome of Hp2-1 individuals, when compared that of Hp1-1 and Hp2-2, is likely to shed light on the mechanism underlying the prognostic effect of Hp. Differences in function between Hp types is likely to result in a dose-dependent effect between genotypes, while a predominant effect of the Hp dimer is likely to result in similar outcomes in Hp2-1 and Hp1-1 individuals, but different from Hp2-2 individuals. So far, small sample sizes have precluded meaningful comparison of the outcome of the heterozygous versus homozygous genotypes. We hypothesised that Hp2-2 individuals are at greater risk of poor short- and long-term outcome after aSAH. A meta-analysis of published studies was performed with the following objectives: (1) to confirm the effect of Hp genotype on outcome after aSAH and (2) to compare the outcome of Hp2-1 individuals with that of Hp1-1 and Hp2-2 and so provide mechanistic insight. In summary, an unfavourable effect of the Hp2-2 genotype on short-term outcome was confirmed. The outcome of Hp2-1 individuals clustered with that of Hp1-1, suggesting that the presence of one Hp1 allele was sufficient to confer protection. Mechanistically, this is in keeping with the hypothesis that the Hp dimer is essential, possibly due to its small size.

## 2. Materials and Methods

**Figure 1:**
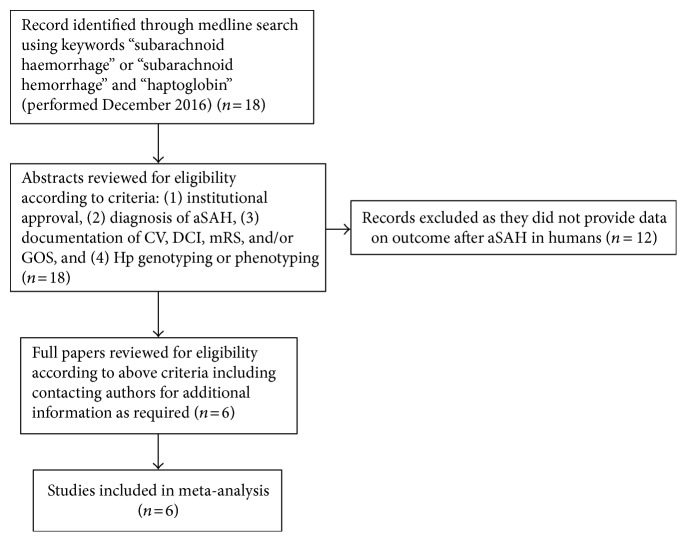
Flow diagram of studies selected for inclusion. If additional information was required, the authors were contacted by email.

Meta-analysis was conducted in accordance with the PRISMA [10] (Supplementary Table 1 available online at https://doi.org/10.1155/2017/6747940) and Cochrane Collaboration guidelines. Data from six studies were included in the meta-analysis (see Figure 1() for search criteria and Table 2 for summary of studies included; all studies were observational) [6, 8, 11–14]. For each individual study, bias was assessed using the Newcastle-Ottawa Scale for quality assessment of nonrandomised studies [15]. This assessment is based on 3 domains (selection, comparability, and outcome) and allows a study to be scored between 0 and 8. Authors BG, DB, and IG independently scored each study; if there was disagreement between scores, an average was taken. All studies included in this analysis scored 5 and were therefore considered to be at low risk of bias (Supplementary Table 2). Although all studies were assessed to be at low risk of bias, the scoring system highlighted the inclusion of higher Fisher grade patients, limited intra-study controls, and short duration of follow-up as potential sources of bias. Tests for funnel plot asymmetry were not performed as the meta-analysis only included 6 studies, in keeping with recommendations from the Cochrane Collaboration. Short- and long-term outcomes were derived from the six studies and analysed separately. Short-term outcome was defined as CV and/or DCI during the inpatient period, as determined by any means, including cerebral angiography, transcranial Doppler ultrasonography, and clinical or radiological evidence of DCI. If both CV and DCI values were available, then DCI data was used in preference due to greater clinical relevance. Both DCI and CV are features occurring in the initial period after SAH, which have a well-demonstrated impact on short-term morbidity, inpatient stay duration, and economic costs, justifying their joint qualification as short-term outcome. Data for short-term outcome was available from five studies (Borsody et al. [11], Galea et al. [8], Ohnishi et al. [14], Leclerc et al. [12], Murthy et al. [13]) and was classified as either present or absent. Long-term outcome was defined as dichotomized mRS or GOS, between one and three months after aSAH. mRS and GOS scores of 0–2 and 4-5, respectively, were considered as favourable outcome, with the rest of the scores being unfavourable. Data for long-term outcome was available from three studies at one month (Murthy et al. [13]) or three months (Ohnishi et al. [14], Kantor et al. [6]). Meta-analysis was conducted in Review Manager (RevMan) v5.3.3. The Mantel-Haenszel (M-H) method for calculating the weighted pooled odds ratio in a fixed effects model was used. Significance was accepted to be present at *p* < 0.05.

## 3. Results

**Figure 2:**
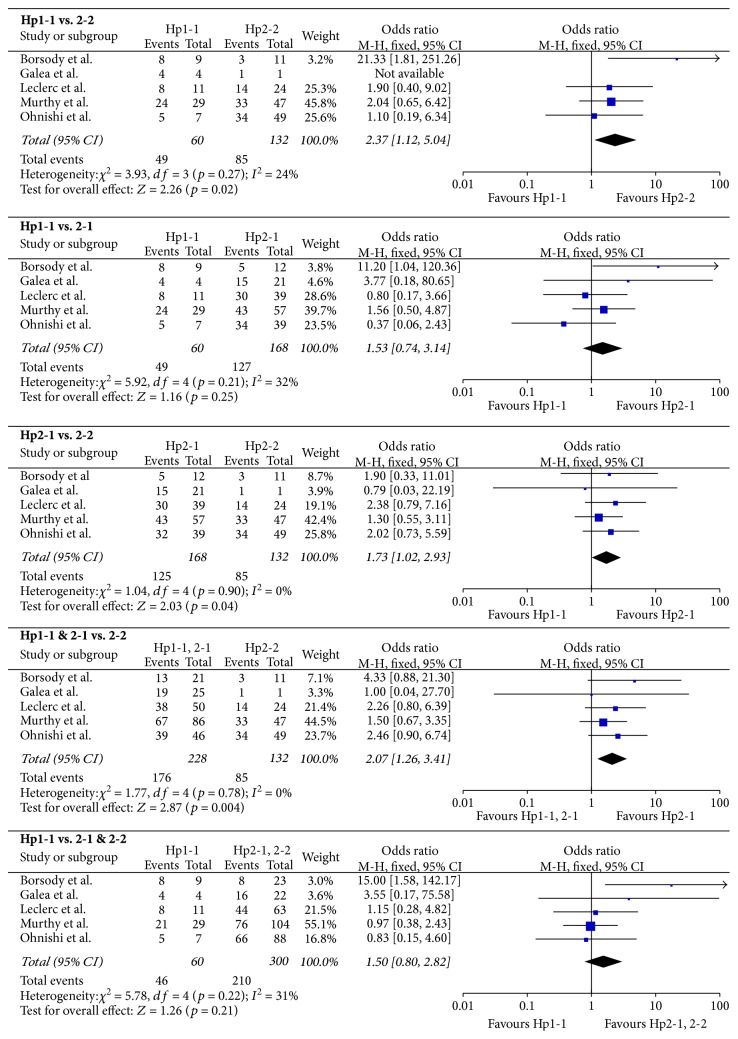
Forest plots for short-term outcome data. Short-term outcome was defined as CV and/or DCI during the inpatient period, as determined by any means, including cerebral angiography, transcranial Doppler ultrasonography, and clinical or radiological evidence of DCI.

**Figure 3:**
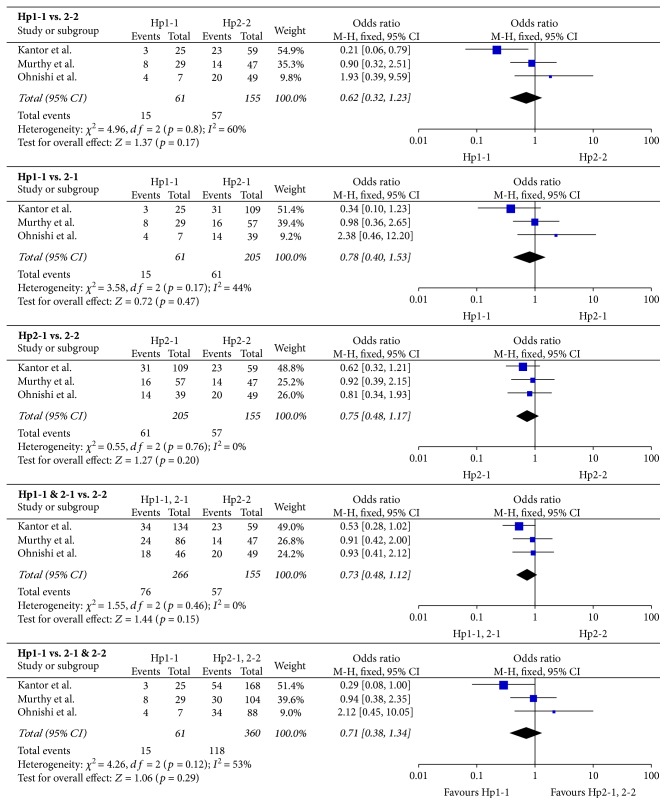
Forest plots for long-term outcome data. Long-term outcome was defined as dichotomized mRS or GOS, between one and three months after aSAH.

553 aSAH patients were included; short-term and long-term outcome data were available for 360 and 421 patients, respectively. Results are presented in Table 3; forest plots are presented in Figures 2 and 3. The Hp2-2 genotype imparted a worse short-term prognosis compared to Hp1-1 (OR = 2.37, 95% CI = 1.12–5.04, *p* = 0.02). The significance of this relationship was increased by including Hp2-1 with Hp1-1 cases (OR = 2.07, 95% CI = 1.26–3.41, *p* = 0.004) and decreased by including Hp2-1 with Hp2-2 cases (OR = 1.50, 95% CI = 0.80–2.82, *p* = 0.21), suggesting that the outcome of Hp2-1 more closely resembled that of Hp1-1 patients. In support of this explanation, the short-term outcome of Hp2-1 patients was significantly different from Hp2-2 (OR = 1.73, 95% CI = 1.02–2.93, *p* = 0.04) but not Hp1-1 patients (OR = 1.53, 95% CI = 0.74–3.14, *p* = 0.25). No effect of Hp genotype on long-term outcome was observed.

## 4. Discussion

Hp can protect against Hb toxicity in a number of ways. First, Hp lowers the redox potential of Hb by binding it. This is achieved by stabilizing the ferryl iron [16] and globin-based amino acid radicals [16, 17], sites within or close to the interface between Hp and Hb [18], preventing these reactive entities from participating in redox reactions. Second, Hp targets Hb for degradation, since Hb-Hp is recognized and cleared by CD163 [1]. Third, Hp induces an anti-inflammatory response (e.g., interleukin-10 secretion [19, 20]), which serves to balance Hb or heme-induced proinflammatory effects (e.g., tumour necrosis factor [21, 22] and interleukin-1 [23] secretion). There is controversy as to whether some of these functions differ between Hp types (Table 1). It is important to note that Hp binding to Hb does not affect its capacity to scavenge nitric oxide [24, 25]. Hence, any nitric oxide-mediated mechanistic basis for differences in vasospasm between Hp genotypes is likely linked to clearance of Hb.

A study of experimental SAH in mice clearly demonstrated differences between Hp1 and Hp2 [26]. Mice only express Hp1 but mice genetically engineered to express Hp2 in place of Hp1 had a poorer outcome after SAH, compared to Hp1 wild-type mice [26]. However, this study did not examine Hp2-1 mice. This meta-analysis has confirmed that the Hp2-2 genotype confers a worse short-term outcome versus Hp1-1 in humans. Moreover, the short-term outcome of Hp2-1 patients clusters with that of Hp1-1 patients, suggesting that the presence of one Hp1 allele is sufficient to confer protection over Hp2.

The findings in the Hp2-1 heterozygous individuals have mechanistic implications. Functional mechanisms such as lowering Hb redox potential, CD163 uptake, or anti-inflammatory effects would be expected to result in dose-dependent differences in outcome between the three genotypes. However, the short-term outcome of Hp2-1 was similar to that of Hp1-1. The common feature amongst Hp1-1 and Hp2-1 individuals is the presence of the Hp dimer, which therefore appears to be important in conferring protection. It is possible that the small size of the dimer facilitates drainage of the Hp-Hb complexes from the brain. Although several studies have shown upregulation of CD163 after intracerebral haemorrhage [27–30], CD163 binding sites are limiting after SAH since free Hp-Hb complexes persist in the cerebrospinal fluid [8, 9], possibly compounded by soluble CD163 shedding [8]. For this reason, drainage of Hp-Hb complexes out of the brain via the glymphatic pathway [7] may be important. There is evidence for a size selectivity in the glymphatic pathway [7] so that molecules with a molecular weight above 200 kDa have reduced clearance. The size of the Hp dimer in complex with Hb would be below this threshold (180 kDa), while Hb in complex with Hp polymers of increasing valency would have higher molecular weights. The small size of the Hp dimer also enables it to enter the brain across the blood-brain barrier while higher-order polymers find it more difficult [31]. Hence, amongst all the Hp forms, the Hp dimer would be able to recycle into and out of the brain with greatest ease, clearing Hb from the brain in the process. In keeping with this explanation, a decrease in serum Hp occurs after aSAH, most marked in individuals with the highest blood-brain barrier disruption [8]. These speculations need to be addressed by experimental work to prove that Hp1-1-Hb complex size impacts on outcome by altering drainage of Hb out of the brain. It is still possible that Hp1/Hp2 differences in lowering Hb redox potential, CD163-mediated Hp-Hb uptake, or anti-inflammatory action could affect outcome in a manner which is not dose-dependent.

The findings of this meta-analysis are important for prognostication in the clinical setting, since Hp 2-2 status clearly reflects a group of individuals who may benefit from closer monitoring within a specialist neurointensive care unit. Hp genotype did not affect long-term outcome in this meta-analysis, despite a clear relationship with short-term outcome. This may be due to several reasons. CV may not be related to long-term outcome and this remains controversial [32]. Due to their relatively crude nature, the GOS and mRS scales may not be sufficiently sensitive to detect differences. Recently, two groups have demonstrated upregulation of neuronal CD163 expression after intracranial haemorrhage in nonhuman models [30, 33, 34]—if this finding is confirmed in humans, neurons in Hp1 individuals may accumulate more intracellular heme/iron, which is toxic [35]. It is possible that the short-term beneficial effects of Hp1-1 on vasospasm are balanced by the long-term deleterious effects of Hp1-1 on neuronal iron accumulation, so that long-term outcome is unaffected overall.

This study has a number of limitations. Long-term outcome combined one- and three-month outcomes; however, a sensitivity analysis excluding the one-month study did not change the finding that Hp genotype did not affect the long-term outcome. Short-term outcome was defined as CV and/or DCI, and these phenomena might not necessarily be equivalent [36]; however, a sensitivity analysis excluding CV showed that DCI-only outcome of Hp2-1 more closely resembled that of Hp1-1 patients: Hp1-1 and Hp2-1 versus Hp2-2 (*p* = 0.02, OR (CI) = 1.9 (1.12–3.22)) and Hp1-1 versus Hp2-1 and Hp2-2 (*p* = 0.83, OR (CI) = 1.08 (0.55–2.14)). We also noted that the majority of participants across all studies had high Fisher grade aSAH, so generalizability of the findings here to other patients with aSAH needs to be approached with caution. Although there was no evidence of significant heterogeneity (Table 3), prognostic factors may still have been distributed asymmetrically amongst studies and/or genotype groups; therefore, an individual patient level data analysis is warranted to weigh up Hp genotype against other prognostic covariates, in determining outcome after aSAH.

## 5. Conclusions

In conclusion, this study confirms the unfavourable effect of the Hp2 allele on short-term outcome after aSAH. It advances the field by showing that the presence of one Hp1 allele is sufficient to counter the unfavourable effect of Hp2. This suggests that the Hp dimer is the structural determinant of the association of the Hp polymorphism with outcome after SAH. Therapies aimed at augmenting Hp may work best if designed to mainly deliver Hp1, rather than elevate Hp nonspecifically, in Hp2-1 and Hp2-2 individuals. Experimental studies are needed to prove this hypothesis.

## Supplementary Material

Supplementary table 1. PRISMA checklist for meta-analysis. Supplementary table 2. Newcastle-Ottawa Assessment Scale scores for studies included in meta-analysis.

## Figures and Tables

**Table 1:** Reported differences between Hp types, relevant to SAH.

Function	No difference between Hp types	Difference between Hp types
Hp expression		Serum Hp1-1 higher than Hp2-2, with Hp2-1 intermediate, in many populations tested, including European (Belgian [37–39], Iceland [40]), East Asian (Japanese [41], Koreans [42]), and African (Black Zimbabweans [43], Gabonese [44], Papuans [45])
Haemoglobin binding: capacity per Hp monomer	(1) Ultrafiltration assay of uncomplexed Hb [46] (2) Mass spectrometry [24]	
Haemoglobin binding: affinity	(1) Surface plasmon resonance [47] (2) Surface plasmon resonance [24] (3) Spectrophotometric signal of Hp-Hp interaction [48]	
Inhibition of Hb-mediated oxidation	(1) Reduction in low density lipoprotein oxidation [24] (2) Reduction in Hb intrinsic redox potential [48] (3) Reduction in Hb auto-oxidation [17]	(1) Hp1-1 better than Hp2-2 at inhibiting protein and lipid oxidation [49] (2) Hp1-1 better than Hp2-2 at inhibiting oxidation of linolenic acid and low-density lipoprotein [46] (3) Hp1-1 better than Hp2-2 at inhibiting lipid peroxidation [47]
Interaction with CD163: affinity		(1) Hp2-2 better than Hp1-1, by surface plasmon resonance and binding of radioiodinated Hp-Hb complexes *in vitro* [1] (2) Hp2-2 better than Hp1-1, by binding of radio-iodinated Hp-Hb complexes *in vitro* [50]
Interaction with CD163: uptake of Hp-Hb complexes	Plasma half-life of Hp-Hb complexes after injection in guinea pigs [24]	(1) Hp2-2 better than Hp1-1, by measurement of free Hb in humans [51] (2) Hp1-1 better than Hp2-2, by uptake of radio-iodinated Hp-Hb complexes in human cells *in vitro* [50]
Effects on inflammation		Binding of Hp1-1-Hb complexes to CD163 results in secretion of the anti-inflammatory cytokine IL-10 [19, 20]

**Table 2:** Summary of studies included in meta-analysis.

Study (year)	Journal	Country	Inclusion/exclusion criteria	Patient number	Short-term outcome^∗^	Long-term outcome^∗^
Leclerc et al. (2015) [12]	Proceedings of the National Academy of Sciences of the United States of America	USA	Inclusion: >18 years, aSAH	Hp 1-1: 11	Clinical deterioration as a consequence of confirmed delayed cerebral ischemia	—
				Hp 2-1: 39		
				Hp 2-2: 24		
Murthy et al. (2016) [13]	Neurosurgery	USA	Inclusion: >18 years, aSAH presenting within 24 h of ictus Exclusion: death on arrival, pregnancy, inability to obtain consent	Hp 1-1: 29	Delayed cerebral ischemia defined as clinical deterioration with radiographic, angiographic or clinical response to treatment with TCD evidence	GOS at 30 days post discharge
				Hp 2-1: 57		
				Hp 2-2: 47		
Kantor et al. (2014) [6]	Journal of Neurosurgery	USA	Inclusion: 18–75 years, angiographic diagnosis of aSAH, Fisher grade 2–4, Caucasian Exclusion: pre-existing neurological disease or deficit	Hp 1-1: 25	—	mRS at 3 months
				Hp 2-1: 109		
				Hp 2-2: 59		
Ohnishi et al. (2013) [14]	Journal of Stroke and Cerebrovascular Diseases	Japan	Inclusion: aSAH treated endovascularly or surgically	Hp 1-1: 7	Delayed cerebral ischemia defined as development of focal neurology of a drop in GCS of 2 points	mRS at 3 months
				Hp 2-1: 39		
				Hp 2-2: 49		
Galea et al. (2012) [8]	Journal of Neurochemistry	UK	Inclusion: SAH requiring external ventricular drainage, paired CSF and serum available Exclusion: external ventricular drain infection	Hp 1-1: 4	Delayed cerebral ischemia defined as development of focal neurology of a drop in GCS of 2 points	—
				Hp 2-1: 21		
				Hp 2-2: 1		
Borsody et al. (2006) [11]	Neurology	USA	Inclusion: >18 years, known date onset SAH, aSAH suspected, Fisher grade 3-4 Exclusion: diseases which affect Hp or development of VS	Hp 1-1: 9	Transcranial Doppler (TCD) evidence of “presumed definite” vasospasm or angiogram evidence of vasospasm both by day 14 after SAH	—
				Hp 2-1: 12		
				Hp 2-2: 11		

^∗^Only outcomes which were available for the meta-analysis are shown.

**Table 3:** Short- and long-term outcome after aSAH.

Comparison groups		*N* Total (group A + B)	Odds ratio for poor outcome Group A/B	*Z*	*p*	Heterogeneity		
Group A	Group B					Chi^2^	df	*I* ^2^
*Short-term outcome*								
Hp2-2	Hp1-1	192 (132 + 60)	2.37 (1.12, 5.04)	2.26	0.02^∗^	3.93	3	24%
Hp1-1	Hp2-1	228 (168 + 60)	1.53 (0.74, 3.14)	1.16	0.25	5.92	4	32%
Hp2-2	Hp2-1	300 (132 + 168)	1.73 (1.02, 2.93)	2.03	0.04^∗^	1.04	4	0%
Hp2-2	Hp1-1 + Hp2-1	360 (132 + 228)	2.07 (1.26, 3.41)	2.87	0.004^∗^	1.77	4	0%
Hp2-1 + Hp2-2	Hp1-1	360 (300 + 60)	1.50 (0.80, 2.82)	1.26	0.21	5.78	4	31%
*Long-term outcome*								
Hp2-2	Hp1-1	216 (155 + 61)	0.62 (0.32, 1.23)	1.37	0.17	4.96	2	60%
Hp1-1	Hp2-1	266 (205 + 61)	0.78 (0.40, 1.53)	0.72	0.47	3.58	2	44%
Hp2-2	Hp2-1	360 (155 + 205)	0.75 (0.48, 1.17)	1.27	0.20	0.55	2	0%
Hp2-2	Hp1-1 + Hp2-1	421 (155 + 266)	0.73 (0.48, 1.12)	1.44	0.15	1.55	2	0%
Hp2-1 + Hp2-2	Hp1-1	421 (360 + 61)	0.71 (0.38, 1.34)	1.06	0.29	4.26	2	53%

Note: ∗ denotes *p* < 0.05; *Z*: test for overall effect.

## References

[1] Kristiansen M., Graversen J. H., Jacobsen C. (2001). Identification of the haemoglobin scavenger receptor. *Nature*.

[2] Boettger L. M., Salem R. M., Handsaker R. E. (2016). Recurring exon deletions in the HP (haptoglobin) gene contribute to lower blood cholesterol levels. *Nature Genetics*.

[3] Andersen C. B., Stødkilde K., Sæderup K. L. (2016). Haptoglobin. *Antioxidants & Redox Signaling*.

[4] Larsson M., Cheng T.-M., Chen C.-Y., Simon J., Janciauskiene S. (2013). Unique assembly structure of human haptoglobin phenotypes 1-1, 2-1, and 2-2 and a predominant Hp 1 allele hypothesis. *Acute Phase Proteins*.

[5] Rivero-Arias O., Wolstenholme J., Gray A. (2009). The costs and prognostic characteristics of ischaemic neurological deficit due to subarachnoid haemorrhage in the United Kingdom. Evidence from the MRC International Subarachnoid Aneurysm Trial. *Journal of Neurology*.

[6] Kantor E., Bayır H., Ren D. (2014). Haptoglobin genotype and functional outcome after aneurysmal subarachnoid hemorrhage. *Journal of Neurosurgery*.

[7] Iliff J. J., Lee H., Yu M. (2013). Brain-wide pathway for waste clearance captured by contrast-enhanced MRI. *The Journal of Clinical Investigation*.

[8] Galea J., Cruickshank G., Teeling J. L. (2012). The intrathecal CD163-haptoglobin-hemoglobin scavenging system in subarachnoid hemorrhage. *Journal of Neurochemistry*.

[9] Durnford A., Dunbar J., Galea J. (2015). Haemoglobin scavenging after subarachnoid haemorrhage. *Acta Neurochirurgica Supplement*.

[10] Liberati A., Altman D. G., Tetzlaff J. (2009). The PRISMA statement for reporting systematic reviews and meta-analyses of studies that evaluate healthcare interventions: explanation and elaboration. *British Medical Journal*.

[11] Borsody M., Burke A., Coplin W., Miller-Lotan R., Levy A. (2006). Haptoglobin and the development of cerebral artery vasospasm after subarachnoid hemorrhage. *Neurology*.

[12] Leclerc J. L., Blackburn S., Neal D. (2015). Haptoglobin phenotype predicts the development of focal and global cerebral vasospasm and may influence outcomes after aneurysmal subarachnoid hemorrhage. *Proceedings of the National Academy of Sciences of the United States of America*.

[13] Murthy S. B., Caplan J., Levy A. P. (2016). Haptoglobin 2-2 genotype is associated with cerebral salt wasting syndrome in aneurysmal subarachnoid hemorrhage. *Neurosurgery*.

[14] Ohnishi H., Iihara K., Kaku Y. (2013). Haptoglobin phenotype predicts cerebral vasospasm and clinical deterioration after aneurysmal subarachnoid hemorrhage. *Journal of Stroke and Cerebrovascular Diseases*.

[15] Stang A. (2010). Critical evaluation of the Newcastle-Ottawa scale for the assessment of the quality of nonrandomized studies in meta-analyses. *European Journal of Epidemiology*.

[16] Cooper C. E., Schaer D. J., Buehler P. W. (2013). Haptoglobin binding stabilizes hemoglobin ferryl iron and the globin radical on tyrosine β145. *Antioxidants & Redox Signaling*.

[17] Pimenova T., Pereira C. P., Gehrig P., Buehler P. W., Schaer D. J., Zenobi R. (2010). Quantitative mass spectrometry defines an oxidative hotspot in hemoglobin that is specifically protected by haptoglobin. *Journal of Proteome Research*.

[18] Andersen C. B., Torvund-Jensen M., Nielsen M. J. (2012). Structure of the haptoglobin-haemoglobin complex. *Nature*.

[19] Guetta J., Strauss M., Levy N. S., Fahoum L., Levy A. P. (2007). Haptoglobin genotype modulates the balance of Th1/Th2 cytokines produced by macrophages exposed to free hemoglobin. *Atherosclerosis*.

[20] Philippidis P., Mason J. C., Evans B. J. (2004). Hemoglobin scavenger receptor CD163 mediates interleukin-10 release and heme oxygenase-1 synthesis: antiinflammatory monocyte-macrophage responses in vitro, in resolving skin blisters in vivo, and after cardiopulmonary bypass surgery. *Circulation Research*.

[21] Figueiredo R. T., Fernandez P. L., Mourao-Sa D. S. (2007). Characterization of heme as activator of Toll-like receptor 4. *The Journal of Biological Chemistry*.

[22] Kwon M. S., Woo S. K., Kurland D. B. (2015). Methemoglobin is an endogenous toll-like receptor 4 ligand-relevance to subarachnoid hemorrhage. *International Journal of Molecular Sciences*.

[23] Greenhalgh A. D., Brough D., Robinson E. M., Girard S., Rothwell N. J., Allan S. M. (2012). Interleukin-1 receptor antagonist is beneficial after subarachnoid haemorrhage in rat by blocking haem-driven inflammatory pathology. *Disease Models & Mechanisms*.

[24] Lipiski M., Deuel J. W., Baek J. H., Engelsberger W. R., Buehler P. W., Schaer D. J. (2013). Human Hp1-1 and Hp2-2 phenotype-specific haptoglobin therapeutics are both effective in vitro and in guinea pigs to attenuate hemoglobin toxicity. *Antioxidants & Redox Signaling*.

[25] Azarov I., He X., Jeffers A. (2008). Rate of nitric oxide scavenging by hemoglobin bound to haptoglobin. *Nitric Oxide*.

[26] Chaichana K. L., Levy A. P., Miller-Lotan R., Shakur S., Tamargo R. J. (2007). Haptoglobin 2-2 genotype determines chronic vasospasm after experimental subarachnoid hemorrhage. *Stroke*.

[27] Cao S., Zheng M., Hua Y., Chen G., Keep R. F., Xi G. (2016). Hematoma changes during clot resolution after experimental intracerebral hemorrhage. *Stroke*.

[28] Dang G., Yang Y., Wu G., Hua Y., Keep R. F., Xi G. (2017). Early erythrolysis in the hematoma after experimental intracerebral hemorrhage. *Translational Stroke Research*.

[29] Liu B., Hu B., Shao S. (2015). CD163/hemoglobin oxygenase-1 pathway regulates inflammation in hematoma surrounding tissues after intracerebral hemorrhage. *Journal of Stroke and Cerebrovascular Diseases*.

[30] Liu R., Cao S., Hua Y., Keep R. F., Huang Y., Xi G. (2017). CD163 expression in neurons after experimental intracerebral hemorrhage. *Stroke*.

[31] Chamoun V., Zeman A., Blennow K. (2001). Haptoglobins as markers of blood-CSF barrier dysfunction: the findings in normal CSF. *Journal of the Neurological Sciences*.

[32] Diringer M. N. (2013). Controversy: does prevention of vasospasm in subarachnoid hemorrhage improve clinical outcome?. *Stroke*.

[33] Chen-Roetling J., Regan R. F. (2016). Haptoglobin increases the vulnerability of CD163-expressing neurons to hemoglobin. *Journal of Neurochemistry*.

[34] Garton T. P., He Y., Garton H. J., Keep R. F., Xi G., Strahle J. M. (2016). Hemoglobin-induced neuronal degeneration in the hippocampus after neonatal intraventricular hemorrhage. *Brain Research*.

[35] Bishop G. M., Robinson S. R. (2001). Quantitative analysis of cell death and ferritin expression in response to cortical iron: implications for hypoxia-ischemia and stroke. *Brain Research*.

[36] Hou J., Zhang J. H. (2013). Does prevention of vasospasm in subarachnoid hemorrhage improve clinical outcome? No. *Stroke*.

[37] Langlois M., Delanghe J., De Buyzere M. (1996). Relation between serum IgA concentration and haptoglobin type. *Clinical Chemistry*.

[38] Louagie H., Delanghe J., Desombere I., De Buyzere M., Hauser P., Leroux-Roels G. (1993). Haptoglobin polymorphism and the immune response after hepatitis B vaccination. *Vaccine*.

[39] Márquez L., Shen C., Cleynen I. (2012). Effects of haptoglobin polymorphisms and deficiency on susceptibility to inflammatory bowel disease and on severity of murine colitis. *Gut*.

[40] Bjornsson E., Helgason H., Halldorsson G. (2017). A rare splice donor mutation in the haptoglobin gene associates with blood lipid levels and coronary artery disease. *Human Molecular Genetics*.

[41] Soejima M., Sagata N., Komatsu N. (2014). Genetic factors associated with serum haptoglobin level in a Japanese population. *Clinica Chimica Acta*.

[42] Park K. U., Song J., Kim J. Q. (2004). Haptoglobin genotypic distribution (including Hp^0^ allele) and associated serum haptoglobin concentrations in Koreans. *Journal of Clinical Pathology*.

[43] Kasvosve I., Gomo Z. A., Gangaidzo I. T. (2000). Reference range of serum haptoglobin is haptoglobin phenotype-dependent in blacks. *Clinica Chimica Acta*.

[44] Fowkes F. J., Imrie H., Migot-Nabias F. (2006). Association of haptoglobin levels with age, parasite density, and haptoglobin genotype in a malaria-endemic area of Gabon. *The American Journal of Tropical Medicine and Hygiene*.

[45] Imrie H., Fowkes F. J., Michon P. (2006). Haptoglobin levels are associated with haptoglobin genotype and alpha+-thalassemia in a malaria-endemic area. *The American Journal of Tropical Medicine and Hygiene*.

[46] Melamed-Frank M., Lache O., Enav B. I. (2001). Structure-function analysis of the antioxidant properties of haptoglobin. *Blood*.

[47] Asleh R., Guetta J., Kalet-Litman S., Miller-Lotan R., Levy A. P. (2005). Haptoglobin genotype- and diabetes-dependent differences in iron-mediated oxidative stress in vitro and in vivo. *Circulation Research*.

[48] Mollan T. L., Jia Y., Banerjee S. (2014). Redox properties of human hemoglobin in complex with fractionated dimeric and polymeric human haptoglobin. *Free Radical Biology and Medicine*.

[49] Bamm V. V., Tsemakhovich V. A., Shaklai M., Shaklai N. (2004). Haptoglobin phenotypes differ in their ability to inhibit heme transfer from hemoglobin to LDL. *Biochemistry*.

[50] Asleh R., Marsh S., Shilkrut M. (2003). Genetically determined heterogeneity in hemoglobin scavenging and susceptibility to diabetic cardiovascular disease. *Circulation Research*.

[51] Na N., Ouyang J., Taes Y. E., Delanghe J. R. (2005). Serum free hemoglobin concentrations in healthy individuals are related to haptoglobin type. *Clinical Chemistry*.

