# Supplementary figures and images for: Haptoglobin Genotype and Outcome after Subarachnoid Haemorrhage: New Insights from a Meta-Analysis
Source: Oxid Med Cell Longev. 2017 Sep 26;2017:6747940. doi: 10.1155/2017/6747940 (PMC5634574; doi:10.1155/2017/6747940)

## Slide 1
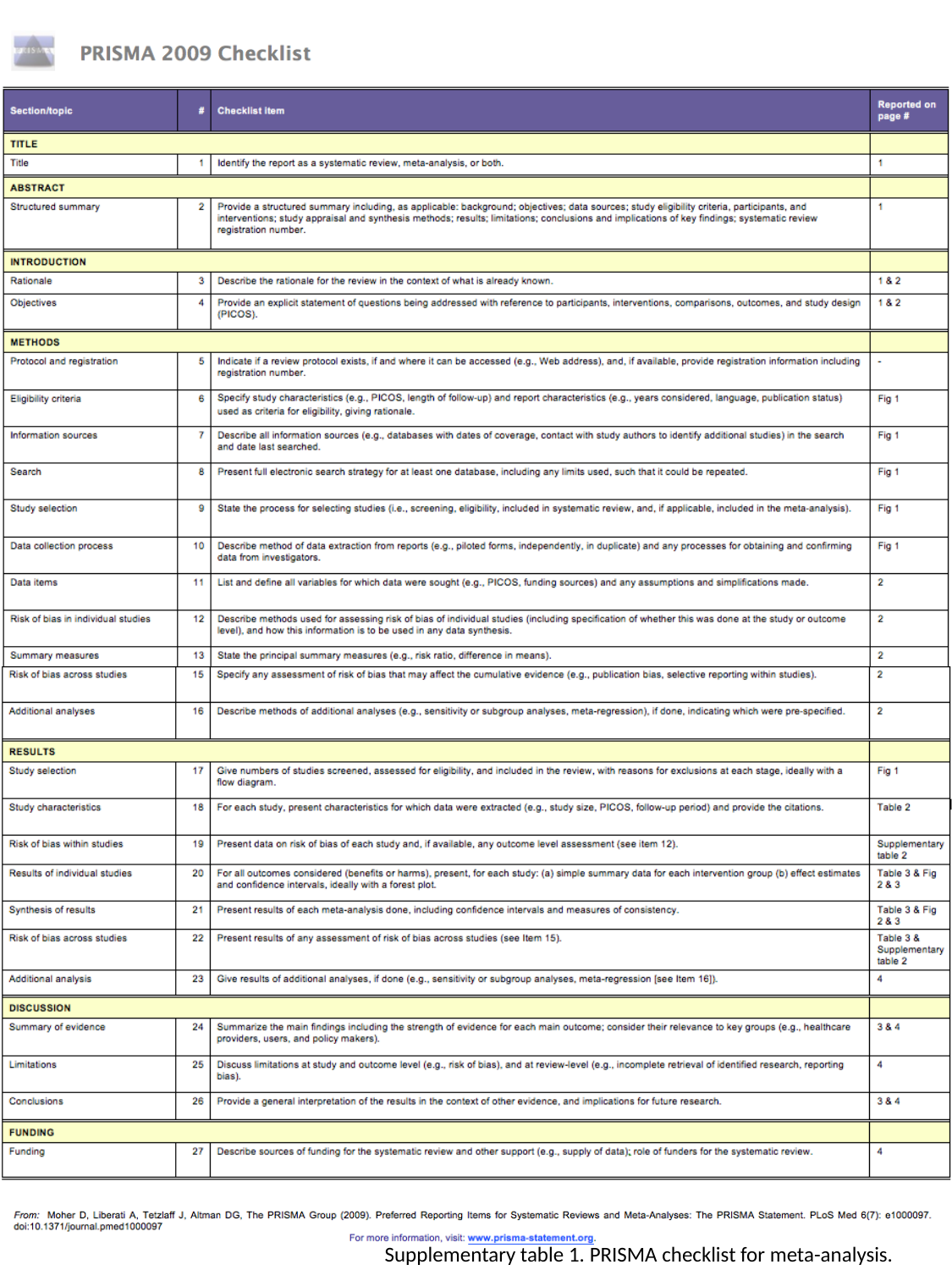

Supplementary table 1. PRISMA checklist for meta-analysis.

Supplement: Supplementary file 1 — Supplementary table 1. PRISMA checklist for meta-analysis. Supplementary table 2. Newcastle-Ottawa Assessment Scale scores for studies included in meta-analysis. [file 6747940.f1.pptx]
