# Supplementary material for: Haptoglobin Genotype and Outcome after Subarachnoid Haemorrhage: New Insights from a Meta-Analysis
Source: Oxid Med Cell Longev. 2017 Sep 26;2017:6747940. doi: 10.1155/2017/6747940 (PMC5634574; doi:10.1155/2017/6747940)
Supplement: Supplementary file 2 [file 6747940.f2.docx]

| Study | Selection | | | | Comparability | Outcome | | | Score |
| --- | --- | --- | --- | --- | --- | --- | --- | --- | --- |
|  | Representativeness of exposed cohort | Selection of the non exposed cohort | Ascertainment of exposure | Demonstration that outcome of interest was not present at start of study | Comparability of cohorts on the basis of the design or analysis | Assessment of outcome | Was follow up long enough for outcomes to occur | Adequacy of follow up of cohorts |  |
| Leclerc et al. | **-** | ***** | ***** | ***** | - | ***** | **-** | ***** | 5 |
| Borsody et al. | **-** | ***** | ***** | * | - | ***** | **-** | ***** | 5 |
| Ohnishi et al. | **-** | ***** | ***** | ***** | - | ***** | **-** | ***** | 5 |
| Kantor et al. | **-** | ***** | ***** | ***** | - | ***** | **-** | ***** | 5 |
| Murthy et al. | **-** | ***** | ***** | ***** | - | ***** | **-** | ***** | 5 |
| Galea et al. | **-** | ***** | ***** | ***** | - | ***** | **-** | ***** | 5 |

Supplementary table 2. Newcastle-Ottawa Assessment Scale scores for studies included in meta-analysis.
